# Exploring the Functional Roles of Telomere Maintenance 2 in the Tumorigenesis of Glioblastoma Multiforme and Drug Responsiveness to Temozolomide

**DOI:** 10.3390/ijms24119256

**Published:** 2023-05-25

**Authors:** Shao-Wei Feng, Zih-Syuan Wu, Yi-Lin Chiu, Shih-Ming Huang

**Affiliations:** 1 Graduate Institute of Medical Sciences, National Defense Medical Center, Taipei 114, Taiwan; heisenber0930@gmail.com; 2 Department of Neurological Surgery, Tri-Service General Hospital, National Defense Medical Center, Taipei 114, Taiwan; 3 Graduate Institute of Life Sciences, National Defense Medical Center, Taipei 114, Taiwan; g8401011@gmail.com; 4 Department of Biochemistry, National Defense Medical Center, Taipei 114, Taiwan; yilin1107@mail.ndmctsgh.edu.tw

**Keywords:** glioblastoma multiforme, temozolomide, telomere maintenance 2, fibroblast growth factor receptor 3, phosphatidylinositol 3-kinase-related kinase

## Abstract

Glioblastoma multiforme (GBM) is a grade IV human glioma. It is the most malignant primary central nervous system tumor in adults, accounting for around 15% of intracranial neoplasms and 40–50% of all primary malignant brain tumors. However, the median survival time of GBM patients is still less than 15 months, even after treatment with surgical resection, concurrent chemoradiotherapy, and adjuvant chemotherapy with temozolomide (TMZ). *Telomere maintenance 2 (TELO2)* mRNA is highly expressed in high-grade glioma patients, and its expression correlates with shorter survival outcomes. Hence, it is urgent to address the functional role of TELO2 in the tumorigenesis and TMZ treatment of GBM. In this study, we knocked down *TELO2* mRNA in GBM8401 cells, a grade IV GBM, compared with *TELO2* mRNA overexpression in human embryonic glial SVG p12 cells and normal human astrocyte (NHA) cells. We first analyzed the effect of TELO2 on the Elsevier pathway and Hallmark gene sets in GBM8401, SVG p12, and NHA via an mRNA array analysis. Later, we further examined and analyzed the relationship between TELO2 and fibroblast growth factor receptor 3, cell cycle progression, epithelial–mesenchymal transient (EMT), reactive oxygen species (ROS), apoptosis, and telomerase activity. Our data showed that TELO2 is involved in several functions of GBM cells, including cell cycle progression, EMT, ROS, apoptosis, and telomerase activity. Finally, we examined the crosstalk between TELO2 and the responsiveness of TMZ or curcumin mediated through the TELO2–TTI1–TTI2 complex, the p53-dependent complex, the mitochondrial-related complex, and signaling pathways in GBM8401 cells. In summary, our work provides new insight that TELO2 might modulate target proteins mediated through the complex of phosphatidylinositol 3-kinase-related kinases in its involvement in cell cycle progression, EMT, and drug response in GBM patients.

## 1. Introduction

Telomere maintenance 2 (TELO2, also known as tel2) was first identified in the telomere length regulation and telomere position in *Saccharomyces cerevisiae* [1,2]. TELO2 directly regulates the stability of phosphatidylinositol 3-kinase-related kinase (PIKK) family members, including ATM (ataxia telangiectasia mutated), ATR (ATM- and rad3-related), and mammalian target of rapamycin (mTOR) [3,4]. Hence, TELO2 is involved in various cellular processes, such as cell proliferation, the biological clock, embryonic development, and the DNA damage response (DDR) [5] for the translation, growth, and autophagic regulation of cells.

There are two TELO2-binding proteins: TELO2-interacting protein 1 (TTI1) and TELO2-interacting protein 2 (TTI2). TTI1 provides a platform on which TELO2 and TTI2 can bind to its central region and C-terminal end, respectively [5,6]. In the absence of TTI1, the expression level of TELO2 is diminished, implying that the two proteins function together. Cells lacking TTI1 exhibit decreased levels of expression for all six PIKKs and result in the disassembly of mTORC1 and mTORC2 because TTI1–TTI2 interacts with mTOR’s FAT (Frap, ATM, TRRAP) and kinase domains [5]. The endogenous mTOR inhibitor DEPTOR antagonizes the TELO2–mTORC1 interaction [7,8]. The stabilization of mTOR by TELO2 overexpression prevents cardiomyocyte cell death by protecting cardiomyocytes against ischemic stimuli [9]. The overexpression of mTOR protects cardiomyocytes from ferroptosis, which is a critical pathophysiological feature in I/R injury [10]. Hence, TELO2 is not only critical for protein stability, but it is also essential for the integrity of mTOR complexes [11]. It should be interesting to address the functional role of TELO2 in sustaining mTOR signaling and tumorigenicity.

CLK-2 is a homologue of TELO2 and serves as a novel player in the nematode nonsenses-mediated mRNA decay (NMD) pathway [12]. TELO2 phosphorylation by casein-kinase 2 (CK2) has been shown to facilitate NMD by increasing the stability of SMG1 via the assembly of PIKKs [13]. Thus, TELO2 and the R2TP (RUVBL1, RUVBL2, PIH1D1, and RPAP3) complex function in NMD alongside DNA damage signaling [6]. The importance of TELO2–TTI1–TTI2 (TTT) assembly is illustrated by its association with syndromic intellectual disability and You–Hoover–Fong syndrome, which are caused by mutations of *TTI1* and *TELO2*, respectively [14,15]. Loss-of-function variants in the human *TELO2* gene have been linked to You–Hoover–Fong syndrome [14,15]. Surprisingly, You–Hoover–Fong syndrome patients lack hallmarks of a DNA repair syndrome, such as cancer and premature aging.

Many studies have revealed that TELO2 might be an oncogenic protein in solid tumors, such as breast cancer and high-grade gliomas [8,11,16,17,18]. Our previous study showed that human high-grade gliomas increase *TELO2* mRNA expression and that overexpression of *TELO2* mRNA expression correlates with shorter survival outcomes, supporting the finding that *TELO2* is an oncogene in human gliomas [16]. Grade III anaplastic astrocytoma and grade IV glioblastoma multiforme (GBM) are both defined as high-grade gliomas with poor survival outcomes by the World Health Organization (WHO) [19,20]. Conventional therapeutic strategies consist of extensive resection and concurrent chemo-radiotherapy. The standard chemotherapy for GBM is temozolomide (TMZ), an alkylation agent that leads to DNA damage [21]. However, the decreasing therapeutic effect and the induced drug resistance of TMZ are mediated through the activation of the phosphatidylinositol-3-kinase (PI3K)/Akt/mTOR signaling pathway [22,23]. The PI3K/Akt and mTOR signaling pathways are two pathways crucial to many aspects of cell growth and survival in physiological as well as in pathological conditions (e.g., cancer) [24,25]. Our previous study showed that four different curcumin analogs, including curcumin, bisdemethoxycurcumin, demethoxy-curcumin, and dimethoxy-curcumin, promote sub-G1 phase, G2/M arrest, apoptosis, and ROS production in human glioma cells [26]. The suppressed activation of the PI3K/Akt/mTOR signaling pathway by curcumin has been reported in many cancer cells, including GBM cells [24,25]. The role of TELO2 in the responsiveness of TMZ and curcumin in GBM cells remains to be addressed.

In this study, we tried to examine the oncogenic roles of TELO2 in GBM cells. First, we overexpressed *TELO2* in two normal human glial cells and silenced *TELO2* expression in GBM8401 cells. Then, we compared TMZ and curcumin with the absence or presence of TELO2 to clarify the involvement of the TTT complex, the p53-dependent complex, the mitochondrial-related complex, and signaling pathways in the therapy of GBM. In summary, our findings might provide new insight on the roles of TELO2 in the cell cycle progression, EMT, and drug response of human high-grade glioma cells.

## 2. Results

**Figure 1:**
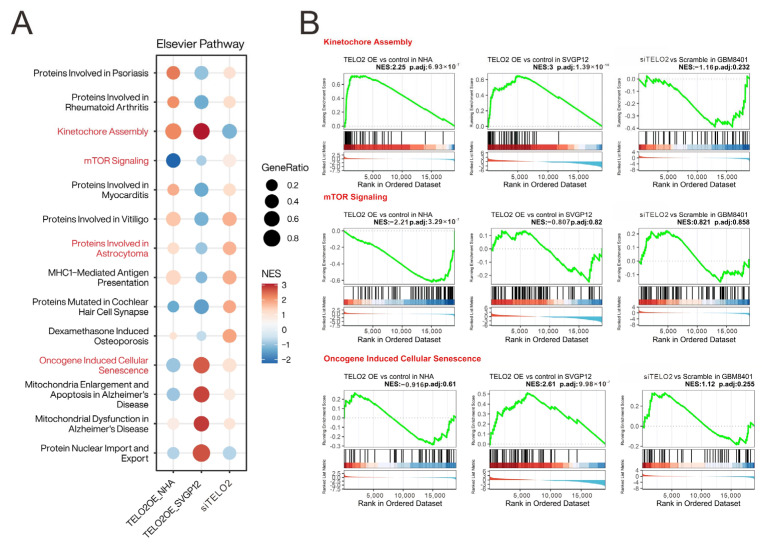
Overall assessment of the enrichment of *TELO2* overexpression and knockdown in disease and signaling pathways. (**A**) Visualization of *TELO2* positive or negative enrichment in Elsevier pathway by compareCluster function in clusterProfiler. GeneRatio represents the proportion of leading-edge genes in the gene set and NES represents the normalized enrichment score. (**B**) The distribution of the enrichment groups in (**A**) is presented as GSEA plots, with the treatment group on the left and the control group on the right.

Our previous study demonstrated that human high-grade gliomas show increased *TELO2* mRNA expression and that the overexpression of *TELO2* mRNA correlates with shorter survival outcomes, suggesting that *TELO2* is an oncogene in human gliomas [16]. Here, we tried to figure out the oncogenic role of TELO2 in tumorigenesis. Hence, we transiently overexpressed *TELO2* mRNA in human glial and astrocyte cells and NHA and SVG p12, respectively, and silenced *TELO2* mRNA expression in GBM8401 cells for the mRNA array analysis. We applied the Elsevier pathway to interpret our experimental data underlying the biology of diseases, responses to drugs, and a wide range of biological processes (Figure 1A). We highlighted the difference between overexpressing and silencing the *TELO2* of normal and GBM cells in the Kinetochore Assembly, mTOR Signaling, Oncogene-Induced Cellular Senescence, and Proteins Involved in Astrocytoma gene set to conduct further analysis based on our previous findings of *TELO2* overexpression in human high-grade gliomas. The results of the gene set enrichment analysis (GSEA) of Kinetochore Assembly, mTOR Signaling, and Oncogene-Induced Cellular Senescence are shown in Figure 1B. 

**Figure 2:**
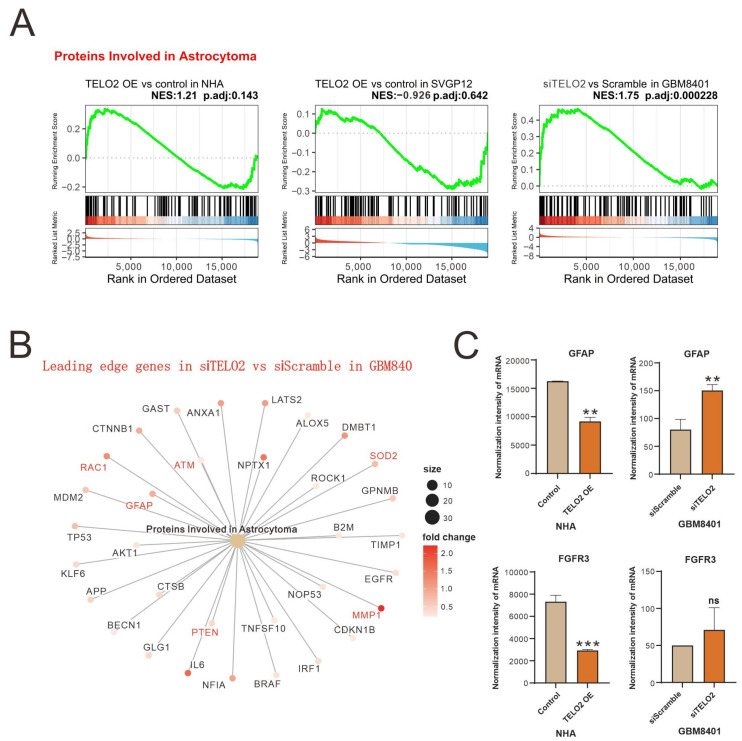
Advanced evaluation of the Proteins Involved in Astrocytoma gene set’s associated genes and fold change. (**A**) GSEA plot showing the enrichment distribution of Proteins Involved in Astrocytoma in each group. (**B**) The fold change of leading-edge gene in siTELO2 vs. siScramble in GBM8401 is presented by cnetplot. (**C**) Bar plot analysis of the difference in mRNA expression of *GFAP* and *FGFR3*, the potential targets of TELO2 expression change. ** *p* < 0.005, *** *p* < 0.001, and ns: not significant.

The results of the GSEA of Proteins Involved in Astrocytoma were further shown in leading-edge genes in knocked-down *TELO2* in GBM8401 cells compared to silencing controls (Figure 2A). Leading-edge genes, including *GFAP* (*glial fibrillary acidic protein*), *RAC1*, *ATM*, *SOD2* (*superoxide dismutase 2*), *MMP2* (*matrix metalloproteinase 2*), and *PTEN* (*phosphatase and tensin homologue*), are highlighted in red font (Figure 2B). We further measured the change in the normalized intensity of *GFAP* and *FGFR3* (*fibroblast growth factor receptor 3*) mRNAs in NHA and GBM8401 cells and found that higher levels of TELO2 might disrupt the expression of *GFAP* and *FGFR3* mRNAs in NHA cells (Figure 2C). 

**Figure 3:**
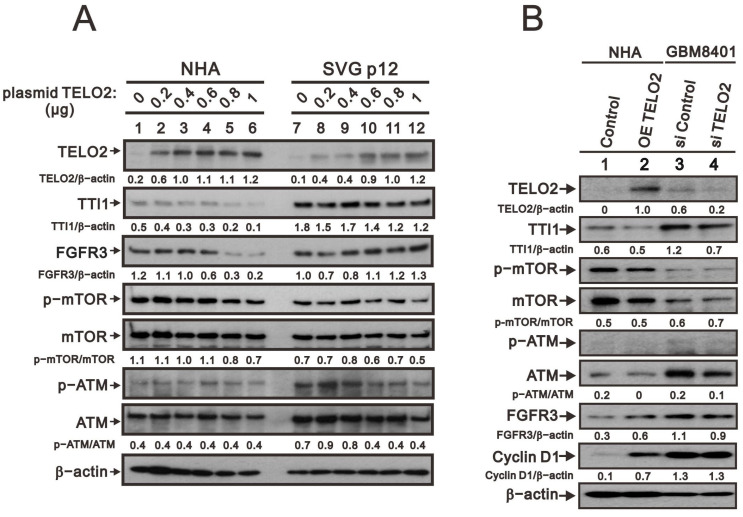
The effects of *TELO2* overexpression in normal glial cell lines and knockdown in glioma cells on protein expression in cells. (**A**) Transfecting plasmid *TELO2* with varying concentrations of 0, 0.2, 0.4, 0.6, 0.8, and 1 µg into NHA and SVGp12 cells to overexpress *TELO2*. (**B**) Overexpression of *TELO2* in NHA cells and knockdown of *TELO2* in GBM8401 cells. The protein levels were analyzed via Western blot analysis. β-actin was used to normalize relative protein expression. The protein bands were quantified through pixel density scanning and evaluated using Image J, version 1.44a (http://imagej.nih.gov/ij/, accessed on 12 May 2023). The ratios of p-protein/total protein and protein/β-actin were plotted in cells.

We further observed in the Western blotting analyses in NHA and SVG p12 cells that TELO2 protein levels were increased with the higher amount of transfected *TELO2* plasmid DNAs. The decreasing trend was consistent with TTI1 and the p-mTOR/mTOR ratio in NHA and SVG p12 cells (Figure 3A). The change trend was inconsistent with FGFR3 and the p-ATM/ATM ratio in NHA and SVG p12 cells. An increasing trend was observed for FGFR3 in SVG p12 cells and the p-ATM/ATM ratio in NHA cells, and a decreasing trend was observed for FGFR3 in NHA cells and the p-ATM/ATM ratio in SVG p12 cells. It was interesting to compare the *TELO2*-overexpressing NHA cells and the silenced *TELO2* expression in GBM8401 cells with the above-mentioned proteins, including TTI1, p-mTOR/mTOR, p-ATM/ATM, and FGFR3 (Figure 3B). The effect of *TELO2* overexpression in NHA cells and the effect of *TELO2* silencing in GBM8401 were examined and confirmed in the Western blotting analysis. Unexpectedly, the trends of TTI1, p-mTOR/mTOR, and p-ATM/ATM were consistent in *TELO2*-overexpressed NHA and *TELO2*-silenced GBM8401 cells. The expression of FGFR3 had a positive relationship with the expression of TELO2 in NHA and GBM8401 cells. In addition, the level of cyclin D1 protein was increased in *TELO2*-overexpressed NHA cells and decreased in *TELO2*-silenced GBM8401 cells, suggesting the oncogenic role of TELO2 protein. 

**Figure 4:**
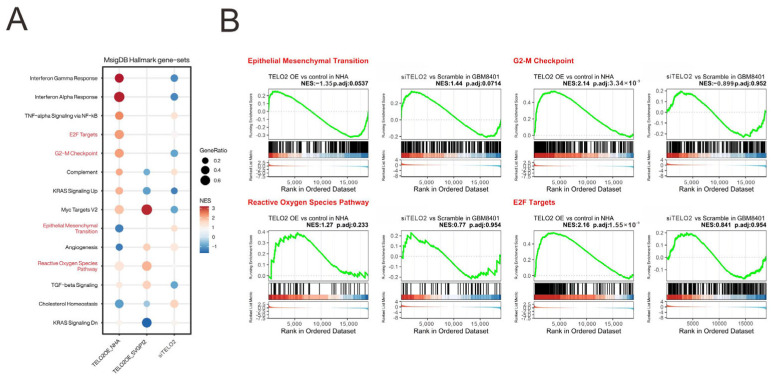
Comprehensive evaluation of the enrichment of *TELO2* overexpression and knockdown in Hallmark gene sets. (**A**) Visualization of *TELO2* positive or negative enrichment in Hallmark gene set using the compareCluster function in clusterProfiler. GeneRatio represents the proportion of leading-edge genes in the gene set and NES represents the normalized enrichment score. (**B**) The distribution of the enrichment groups in (**A**) is presented as GSEA plots, with the treatment group on the left and the control group on the right.

**Figure 5:**
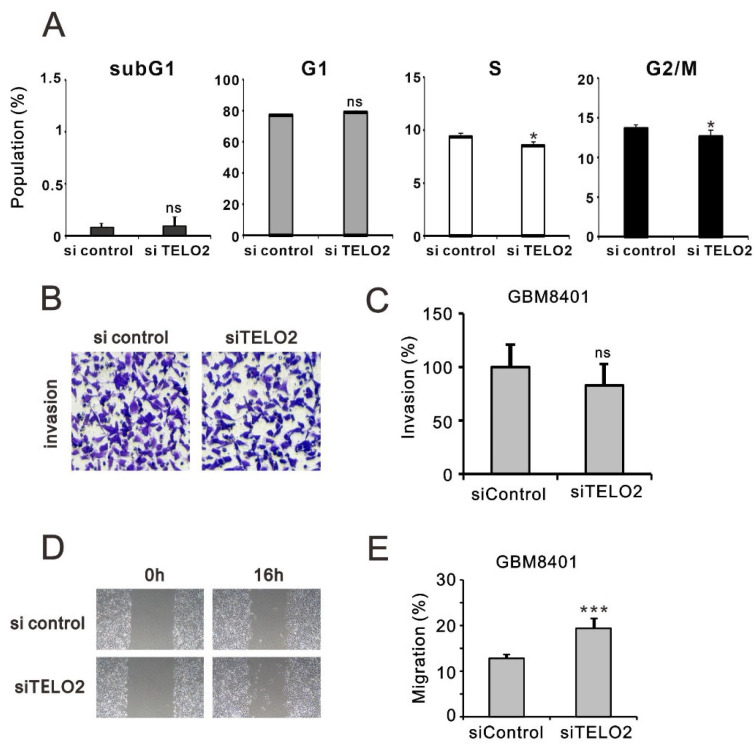
Alterations in glioma cell cycle profiles, invasion, and migration resulting from *TELO2* knockdown. (**A**) GBM8401 cells were stained with propidium iodide (PI) and analyzed using flow cytometry. (**B**,**C**) Evaluating the effect of *TELO2* gene knockdown on the invasiveness of GBM8401 cells. (**D**,**E**) Assessing the effect of *TELO2* gene knockdown on the migration capacity of GBM8401 cells. B Bars depict the mean ± SD of three independent experiments. Student’s *t*-tests were analyzed and compared with siControl. * *p* < 0.05, *** *p* < 0.001, and ns: not significant.

We analyzed and highlighted the difference between overexpressing and silencing *TELO2* in normal and GBM cells in Hallmark gene sets (Figure 4A) involved in oncogenic functions, including E2F targets and G2-M checkpoints (related to the cell cycle), epithelial–mesenchymal transient (EMT), and the reactive oxygen species (ROS) pathway, via GESAs (Figure 4B). We further verified the effect of TELO2 on the cell cycle profile via flow cytometry analysis in GBM8401 cells. We observed significantly decreased populations of S and G2/M phases in *TELO2*-silenced GBM8401 cells (Figure 5A). No significant change was observed in the populations of subG1 and G1 phases. In addition to cell cycle progression, we also examined the effects of TELO2 on the EMT, including the migration and invasion of GBM8401 cells. Our data showed that TELO2 played a minor effect on invasion (Figure 5B,C) but significantly suppressed the migration rate of GBM8401 cells (Figure 5D,E). 

**Figure 6:**
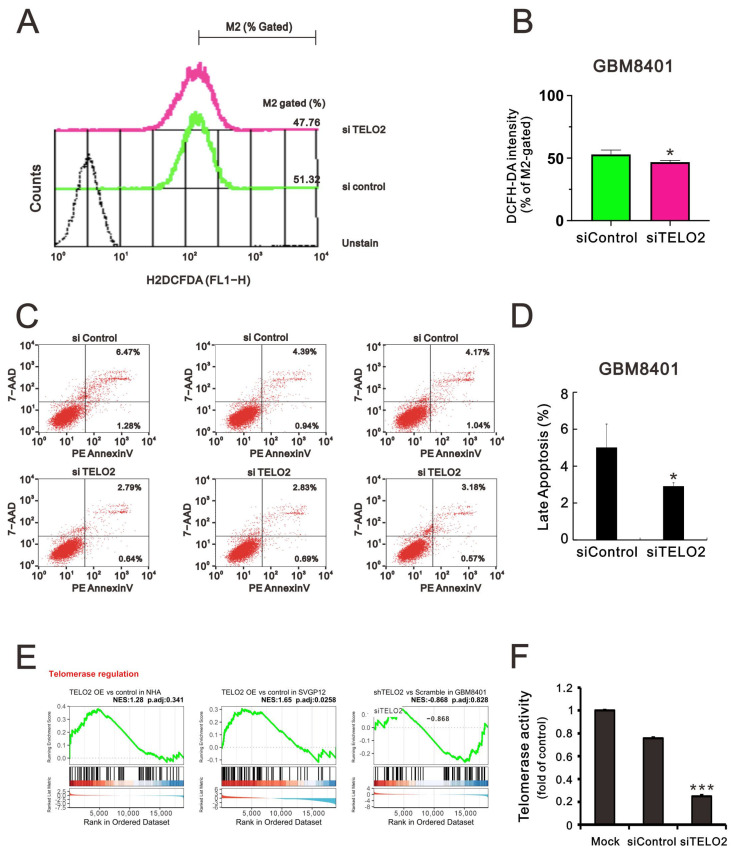
Investigating the impact of *TELO2* knockdown on cellular ROS, apoptosis, and telomerase activity in glioma cells. (**A**,**B**) Intracellular ROS levels were assessed using DCFH-DA staining with flow cytometry. (**C**,**D**) After *TELO2* knockdown in GBM8401 cells, apoptosis markers labeled by PE-Annexin V and 7-AAD were analyzed using flow cytometry. (**E**) GSEA plot showing the enrichment distribution of telomerase regulation in each group. (**F**) The telomerase activity of Mock, siControl, and siTELO2-treated GBM8401 cells was evaluated using a TRAP assay kit. Bars depict the mean ± SD of three independent experiments. Student’s *t*-tests were analyzed and compared with siControl. * *p* < 0.05, *** *p* < 0.001.

The TTT complex not only regulates the levels of ATM, ATR, and other PIKKs, but it also compromises the interaction between ATR and ATR interacting protein [5]. ATM and ATR are activated by oncogenic stresses, suggesting that cancer cells may rely on DNA damage response pathways to survive genomic instability [27]. Hence, we sought to determine the effect of TELO2 on ROS and apoptotic stress in GBM8401 cells. We applied flow cytometry analysis with DCFH-DA fluorescent dye to measure cytosolic ROS. Our data showed that the dye’s peak had a left shift in *TELO2*-silenced GBM8401 cells (Figure 6A,B). In the Annexin-V apoptotic analysis, the apoptotic populations of early and late-stage glioblastomas were reduced in *TELO2*-silenced GBM8401 cells (Figure 6C,D). These findings suggest that TELO2 might play a role in the cellular stress, including ROS and apoptosis. TELO2 was required in telomere length regulation and telomere position. Hence, the results of the GSEA of telomerase regulation were analyzed, and we further examined the telomerase activity of *TELO2*-silenced GBM8401 cells (Figure 6E,F). Our data showed that the telomerase activity was significantly decreased when TELO2 in GBM8401 cells was silenced compared with the silenced control and mock cells.

TMZ is the standard chemotherapy for GBM, and its drug resistance might be mediated through the activation of the PI3K/Akt/mTOR signaling pathway [21]. Curcumin was shown to promote the sub-G1 phase, G2/M arrest, apoptosis, and ROS production in our previous study on human glioma cells [26]. The suppressed activation of the PI3K/Akt/mTOR signaling pathway by curcumin has been reported in many cancer cells, including GBM cells [24,25]. Hence, we applied TMZ and curcumin to address the role of TELO2 in the treatment of GBM8401 cells. We first examined the silencing efficacy of *TELO2* using RT–PCR and Western blotting analysis. The downregulations of *TELO2* mRNA and protein expressions were confirmed in *TELO2*-silenced GBM8401 cells when cells were treated with TMZ or curcumin. The components of the TTT complex (namely, *TTI1* and *TTI2* mRNAs) were checked, and TELO2 was positively correlated with the vehicle and TMZ for *TTI1* mRNA and TMZ and curcumin for *TTI2* mRNA. TTI1 proteins were positively correlated with TELO2 proteins in GBM8401 cells treated with vehicle, TMZ, and curcumin.

**Figure 7:**
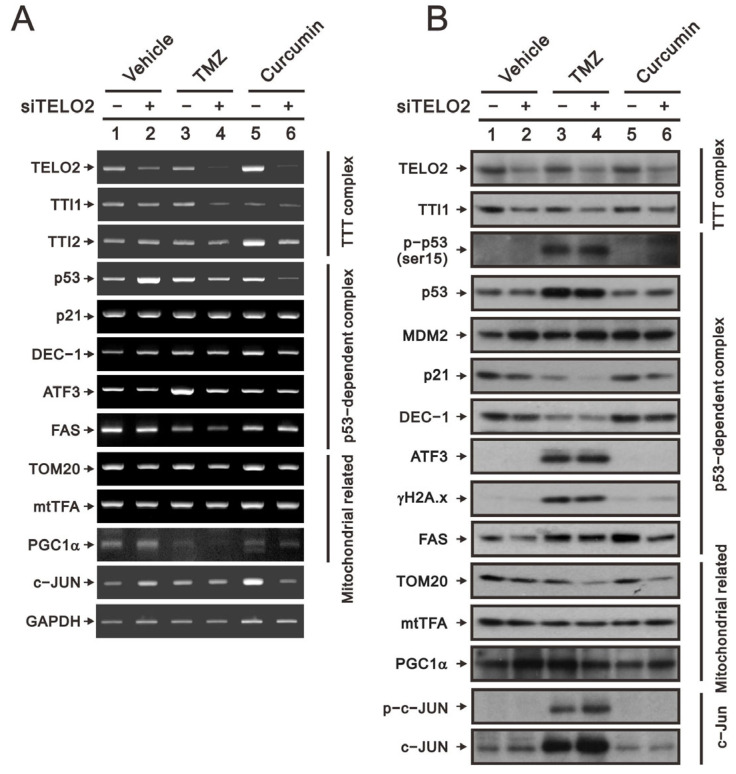
Evaluating the effect of *TELO2* knockdown followed by the treatment of TMZ or curcumin on the mRNA and protein expression in GBM cells. GBM8401 cells were subjected to *TELO2* knockdown and then treated with either 50 µM TMZ for 48 h or 2.5 µM curcumin for 24 h. GBM-treated cells were extracted and analyzed for mRNAs (**A**) and proteins (**B**).

In response to DNA damage, the initial activation trigger is the phosphorylation of p53 at serine 15 (S15) by three kinases in the family of PIKKs: ATM, ATR, and the DNA-dependent protein kinase catalytic subunit [28]. We observed the silencing effect of *TELO2* on the induction of *p53* mRNA in GBM8401 cells. No effect of TMZ, curcumin, or TELO2 on *p21* or *DEC1*, two p53 target genes, was observed. The protein levels of p21 and DEC1 were decreased by TMZ, and both proteins were further decreased in TELO2-silenced GBM8401 cells. The induced *ATF3* mRNA and the *FAS* mRNA repressed by TMZ treatment were further suppressed by the silenced *TELO2* in GBM8401 cells (Figure 7A). TMZ increased the endogenous and phosphorylated p53 residue serine 15, even in the presence of MDM2 proteins inducible by the downregulation of TELO2 in GBM8401 cells (Figure 7B). However, a sufficient amount of p53 failed to be induced in *TELO2*-silenced GBM8401 cells because of the increasing amount of p53-target MDM2 proteins, resulting in subsequent p53 protein degradation. The levels of the ATF3 and γH2A.x proteins were induced by TMZ, and no further effect was induced by TELO2 in GBM8401 cells. The levels of FAS proteins were induced by TMZ and curcumin, which were suppressed in *TELO2*-silenced GBM8401 cells.

Tom20 is a mitochondrial inner protein, and its mRNA and protein were decreased in *TELO2*-silenced GBM8401 cells whether treated with TMZ or curcumin. mtTFA and PGC-1α are important transfection factors and coactivators, respectively, for mitochondrial biogenesis. The mRNA and protein of mtTFA had no apparent effect following exposure to TELO2, TMZ, and curcumin in GBM8401 cells (Figure 7A,B). The mRNA of *PGC-1α* was increased in *TELO2*-silenced GBM8401 cells but was decreased by TMZ and curcumin. We observed a further decrease with TMZ and curcumin in *TELO2*-silenced GBM8401 cells. The protein level of PGC-1α was increased by silenced *TELO2* and curcumin in *TELO2*-silenced GBM8401 cells. The induction of PGC-1α protein by TMZ was further decreased in *TELO2*-silenced GBM8401 cells. *c-Jun* mRNA was increased by silenced *TELO2* and curcumin in *TELO2*-silenced GBM8401 cells. The protein levels of c-Jun and p-c-Jun were induced by TMZ and further increased in *TELO2*-silenced GBM8401 cells.

**Figure 8:**
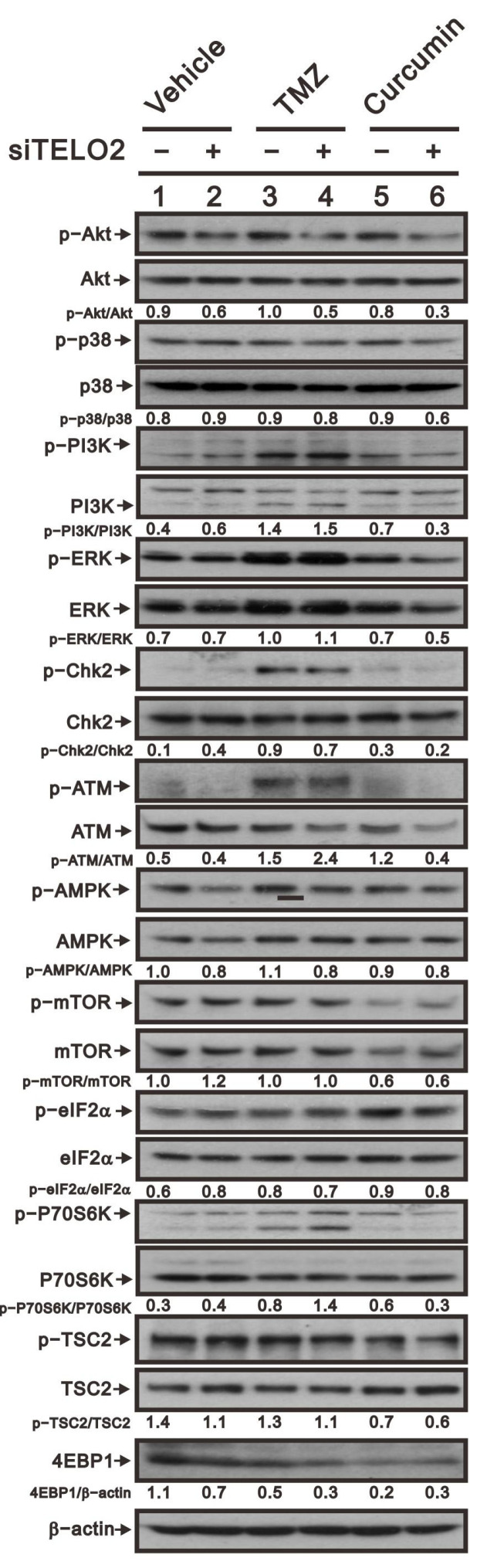
Evaluating the effect of *TELO2* knockdown followed by the treatment of TMZ or curcumin on the signaling pathways in GBM cells. GBM8401 cells were subjected to *TELO2* knockdown and then treated with either 50 µM TMZ for 48 h or 2.5 µM curcumin for 24 h. Proteins were extracted and indicated signaling pathways were analyzed using Western blot analysis. The protein bands were quantified through pixel density scanning and evaluated using Image J, version 1.44a (http://imagej.nih.gov/ij/, accessed on 1 February 2023). The ratios of p-protein/total protein and protein/β-actin were plotted in GBM8401 cells.

Activating receptor tyrosine kinases stimulates mTORC1 signaling by stimulating the translational machinery of the PI3K/AKT and Ras/ERK pathways [29,30]. mTORC1 modulates mRNA translation by promoting the phosphorylation of downstream substrates, including the eukaryotic translation initiation factor (eIF), 4E binding proteins (4E-BPs), and ribosomal S6 kinases (S6Ks), the latter having phosphorylation substrates of their own, including eIF4B, ribosomal protein S6 (rpS6), and programmed cell death protein 4 [31]. Here, we examined the phosphate status of Akt, p38, pI3K, Erk, Chk2, ATM, AMPK, mTOR, eIF2a, p70S6K, and TSC to elucidate the functional roles of TELO2, TMZ, and curcumin in GBM8401 cells (Figure 8). Our data showed that the ratios of p-p38/p38, p-pI3K/pI3K, p-Chk2/Chk2, p-mTOR/mTOR, p-eIF2α/eIF2α, and p-p70S6K/p70S6K were increased, and the ratios of p-Akt/Akt, p-ATM/ATM, p-AMPK/AMPK, and p-TSC-TSC were decreased by the silencing of *TELO2*. However, no effect on p-mTOR/mTOR or the downregulation of p-TSC2/TSC2 was shown, while TMZ treatment increased other signaling pathways in GBM8401 cells. For curcumin, the ratios of p-p38/p38, p-pI3K/pI3K, p-Chk2/Chk2, p-ATM/ATM, p-eIF2α/eIF2α, and p-p70S6K/p70S6K were increased, and p-Akt/Akt, p-AMPK/AMPK, p-mTOR/mTOR, and p-TSC2-TSC2 were decreased by curcumin. The consistent effects of TELO2 in the treatments of TMZ and curcumin were the decreasing ratios of p-AMPK/AMPK and p-TSC2-TSC2 in GBM8401 cells. The effect on the expression of 4E-BP1 was decreased when the *TELO2* in the cells was knocked down or the cells were treated with TMZ or curcumin.

## 3. Discussion

TELO2 binds with PIKK family members, leading to the translation, growth, and autophagic regulation of cells [3,4]. TELO2 is not only critical for protein stability, but it is also essential for the integrity of mTOR complexes. Many studies have shown that TELO2 might play an oncogene role in solid tumors [11,16,17,18]. Our previous study showed that increased *TELO2* mRNA expression in human high-grade gliomas correlates with shorter survival outcomes, suggesting that *TELO2* is an oncogene in human gliomas [16]. Grade III anaplastic astrocytoma and grade IV GBM are both defined as high-grade gliomas by the WHO. In this study, we compared knocked-down *TELO2* mRNA in GBM8401 cells, a grade IV GBM, with *TELO2* mRNA-overexpressing SVG p12 and NHA cells. GBM is a type of cancer that starts as a growth of cells called astrocytes that support nerve cells in the brain or spinal cord. SVG p12 was established by transfecting cultured human fetal glial cells. NHA cells are glial cells that can be used to study the function of the central nervous system and how neural cells interact. Based on the original source of NHA and our current results, NHA might be a better control cell to study the functional role of TELO2 in GBM. In this study, we compared knocked-down *TELO2* mRNA in GBM8401 cells, a grade IV GBM, with *TELO2* mRNA overexpression in human embryonic glial SVG p12 cells and NHA cells. We first analyzed the effect of TELO2 on the Elsevier pathway and Hallmark gene sets in GBM8401, SVG p12, and NHA cells via the mRNA array analysis. Later, we further examined and analyzed the relationship between TELO2 and FGFR3, cell cycle progression, EMT, ROS, apoptosis, and telomerase activity. Our data showed that TELO2 was involved in the cell cycle progression, EMT, ROS, apoptosis, and telomerase activity of GBM cells. Finally, we examined the crosstalk between TELO2 and the responsiveness of TMZ or curcumin mediated through the TTT complex, the p53-dependent complex, the mitochondrial-related complex, and signaling pathways in GBM8401 cells. In summary, our work provides new insight into how TELO2 might modulate target proteins mediated through the complex of PIKKs in its the involvement in cell cycle progression, EMT, and drug response in GBM patients.

The activation of mTORC1 occurs when cells lose the PTEN, neurofibromatosis 1, LKB1, or p53 tumor suppressors [32,33,34]. The mTORC1 pathway regulates growth through downstream effectors, such as the regulators of translation 4EBP1 and S6K1. In addition to its role in promoting protein synthesis, S6K1 represses the PI3K–Akt pathway by inhibiting IRS1 (insulin receptor substrate 1) and IRS2 expression. Hence, an active mTORC1 pathway can suppress PI3K–Akt signaling. The TTT complex has been proposed to recognize newly synthesized PIKKs and to deliver them to the R2TP complex and the heat shock protein 90 chaperone, thereby supporting their folding and assembly [35]. TELO2 might mediate through the conformational changes of PIKKs in response to various stimuli. In addition to that, TELO2 is a common stabilizer of PIKKs, so TELO2 might also serve as a scaffold protein that mediates signal transduction from PIKKs to their target proteins [6,18]. The disruption of the TELO2-binding ability of individual PIKKs would allow cells to rapidly downregulate specific signaling pathways. Here, we examined whether the responses of signaling pathways by TMZ and curcumin could be disrupted by the level of TELO2 in cells. Our findings suggested that the effectiveness of this TELO2-driven therapy should be further confirmed for the functions of TELO2 in normal or cancer cells.

The PI3K/AKT/mTOR pathway is an intracellular signaling pathway important in regulating the cell cycle, cellular quiescence, proliferation, cancer, and longevity [24,25,36]. Activation of the PI3K/Akt and ERK signaling pathways by FGFR promotes cellular growth and EMT in many aggressive forms of cancer. The overexpression of FGFR3 has been associated with several types of cancer, including bladder cancer, non-small cell lung cancer, and oral cancers [37,38]. A subset of GBM harbors oncogenic fusions that join the members of FGFR3 and FGFR1 tyrosine kinases to the transforming acidic coiled-coil (TACC) proteins TACC3 and TACC1, respectively, and it is hoped that the inhibition of FGFR could be a valuable therapeutic option for this subgroup of deadly types of brain cancer or other cancers [17]. Our current findings also suggested that TELO2 might play oncogenic functions mediating the cell cycle profile, EMT, and PI3K–AKT pathways in GBM8401 cells. Our data showed that *TELO2* downregulated the expression of *FGFR3* in NHA and GBM8401 cells, which is consistent with the idea that genes negatively related to FGFR3 demonstrated tumor-related functions, such as mitosis and cell cycle, and FGFR3 correlated with relatively differentiated cellular function in gliomas. In addition to *FGFR3*, these leading genes, such as *SOD2* and *MMP2*, have potential functional roles in ROS-related signaling activations and EMT. A recent study demonstrated that *TELO2* promotes the incorporation of TRRAP into both SAGA and TIP60 complexes, and it regulates the expression of a large fraction of TRRAP-dependent genes [39]. TTT has an important role in sustaining the activities of these oncogenic transcription factors, including c-MYC and E2Fs, in colorectal cancer cells [39]. These transcription factors might facilitate the application of TELO2-targeting drugs in the development of cancer cell vaccines that can prevent cancer recurrence. One supporting study showed that ivermectin (IVM), derived from a mixture of avermectins B1a and B1b, bound to TELO2 to inhibit PIKKs and reduce cytoplasmic β-catenin levels [40]. Hence, TELO2 might be a druggable target for human diseases involving abnormalities of the Wnt/β-catenin pathway and PIKKs, including mTOR. It might be interesting to address whether the regulation of FGFR3 or other proteins by TELO2 is meditated through the stability of PIKKs or transcription factors in cells.

Even after receiving maximum treatment for GBM, including surgical treatment, radiotherapy, and chemotherapy, patients only have a median survival time of 15 months [21,23]. The most common reason why GBM treatment fails is TMZ resistance. MGMT (O^6^-methylguanine-DNA methyltransferase) plays the most important role in TMZ chemoresistance. In addition to the epigenetic regulation of MGMT, irradiation-survivor GBM cells upregulate the expression of DDR-related genes, such as *ATM*, *ATR*, and *MGMT*, and have better DNA repair capacity [41]. The knockdown of *TELO2* resulted in a decrease in PIKKs at the protein level, mediated through the stability of PIKKs. An interesting issue to address is whether MGMT as well as PIKKs (ATM and ATR) are regulated by TELO2 at the protein level or not. The overexpression of TELO2 might be one of reasons for the resistance of some GBM subpopulations to TMZ treatment.

With the advancements that molecular characteristics have played in predicting the prognosis and treatment of GBM [42], identifying the potential therapeutic target is one of the unmet medical needs. *TERT* is a gene that encodes the enzyme telomerase reverse transcriptase, which is involved in maintaining the length of telomeres. Approximately 60–80% of GBM patients were identified as carrying mutations of the *TERT* promoter region, which leads to increased telomerase activity and enables replicative immortality [43]. A defining feature of a small fraction of secondary GBM is the activation of a telomerase-independent alternative lengthening of telomeres (ALT) mechanism, which is driven by homologous recombination machinery [44,45]. TELO2 was first identified in the telomere length regulation and telomere position in *Saccharomyces cerevisiae* [1,2]. In addition to telomere length regulation, TELO2 also directly regulates the stability of PIKK family members for its functions in sustaining mTOR signaling and tumorigenicity. Hence, it is an urgent issue to address and measure the length of telomere of GBM and to further identify the mechanisms by which it might be involved in the *TERT* promoter mutation or/and ALT. The major limitation of our current study is that it is an in vitro study. GBM8401 cells were established and characterized by Dr. Lee of our medical center in 1988 [46]; hence, our team will further characterize different molecular characteristics of patient-derived GBM cell lines to understand the functional role of TELO2 in the status of telomere length differentially regulated by the *TERT* promoter mutation, ALT pathways, or other molecular targets verified in GBM patients. 

## 4. Materials and Methods

### 4.1. Cell Culture and Reagents

NHA (normal human astrocyte) cells were purchased from Lonza Bioscience (CC-2565) (Bend, OR, USA) and maintained in 4.5g/mL glucose Dulbecco’s modified Eagle’s medium (DMEM; Corning, NY, USA) containing 1% N-2 Supplement (Thermo Fisher Scientific, Waltham, MA, USA). SVGp12 (simian virus 40-immortalized human fetal astrocyte) cells were purchased from ATCC (CRL-8621) (Manassas, VA, USA) and cultured in Eagle’s Minimum Essential media (Thermo Fisher Scientific). GBM8401 (glioblastoma multiforme) cells were established and characterized by the team of Dr. Lee [46] and cultured in 4.5g/mL glucose DMEM. All culture media were supplemented with 10% fetal bovine serum (Thermo Fisher Scientific) and penicillin–streptomycin (Thermo Fisher Scientific). The cells were incubated at 37 °C under 5% CO_2_ and 95% air. Temozolomide, curcumin, 2′,7-dichlorofluorescein diacetate (DCFH-DA), and propidium iodide (PI) were obtained from Sigma Aldrich (Sigma Aldrich; St. Louis, MO, USA).

### 4.2. siRNA and Transient Plasmid Transfection

*TELO2* siRNA and siScramble were purchased from Dharmacon (Lafayette, CO, USA). The full-length PCR fragment of human *TELO2* was amplified, cut with EcoRI/XhoI, and cloned into the pSG5.HA expression vector. pSG5.HA–*TELO2* fusion expression clones were successfully obtained. Cells were seeded and incubated at 37 °C under 5% CO_2_ for 24 h. On the following day, the transfection of cells was performed using DharmaFECT 1 transfection reagent (Dharmacon) in accordance with the manufacturer’s instructions, with either 25 nM siTELO2 or siScramble used for knockdown or 0.5 µg pSG5.HA-*TELO2* or empty vector for overexpression.

### 4.3. mRNA Expression Profiling

Parental, *TELO2*-overexpressed, or siRNA-interfered cell lines were pretreated, and total RNA was extracted using TRIzol RNA Isolation Reagent according to the manufacturer’s instructions. The obtained total RNA (*n* = 2) was sent to Phalanx Biotech Group (Hsinchu, Taiwan) for a gene expression profiling service using the HOA OneArray method and HmiOA v5. The amount and purity of RNA were assessed by NanoDrop ND-1000 (ThermoFisher Scientific Inc., Waltham, MA, USA), and the passing criteria were determined to be A260/A280 ≥ 1.8 and A260/A230 ≥ 1.5, indicating acceptable purity of RNA. RNA integrity values (RIN) were determined using an Agilent RNA 6000 Nano detector (Agilent Technologies, Waldbronn, Germany), and the qualifying criterion for RIN values was determined to be ≥6, indicating acceptable RNA integrity. Data analysis was processed by the Rosetta Resolver^®^ system (Rosetta Biosoftware v7.2, Seattle, WA, USA).

### 4.4. Gene Set Enrichment Analysis (GSEA)

To simultaneously assess the differences in biological function and disease pathways corresponding to trends under *TELO2* overexpression or siRNA interference with expression, gene set enrichment analysis (GSEA) was applied for comparison of each subgroup (*TELO2* overexpression vs. control in NHA, *TELO2* overexpression vs. control in SVGP12, and siTELO2 vs. siScramble in GBM8401). Differentially expressed genes (DEGs) were defined using edgeR for each subgroup comparison [47]. As input to edgeR, normalized counts from the microarray analysis were used. All gene expression fold changes were pre-sorted based on the edgeR results to generate a list of genes, which were then analyzed using the “GSEA” function in the clusterProfiler package (v4.6) [48]. For the gene sets, the Elsevier pathway collection was downloaded from Enrichr [49,50], which contains 1721 signaling and disease pathway signatures. In addition, Hallmark gene sets were downloaded from MsigDB, which contains 50 cancer-related major biological functions [51]. The analysis results were plotted using the functions gseaplot2 and compareCluster in clusterProfiler. Among them, compareCluster uses a list of genes generated from the three subgroups of edgeR results, which are analyzed using the “fun = GSEA” setting. Benjamini–Hochberg-defined false discovery rates (FDRs) are shown in the dot plot, and q-values less than 0.25 are considered significantly enriched. For the Proteins in Astrocytoma gene set, the association distribution and fold change of the leading gene in siTELO2 vs. scramble in GBM8401 was plotted using cnetplot.

### 4.5. Western Blot

Protein extraction was performed by lysing the cells in RIPA (radio-immunoprecipitation assay) buffer. The protein concentration was determined using the DC protein assay, and equal amounts of protein were loaded onto SDS-PAGE gels. After electrophoresis, the proteins were transferred onto PVDF membranes and blocked with 5% nonfat milk in TBST for 1 h. The primary antibodies were added and incubated overnight at 4 °C with shaking. The membranes were then washed and incubated with secondary antibodies for 1 h at room temperature. The primary antibodies of TTl1, FGFR3, α-actin, p53, p21, ATF3, FAS, TOM20, mtTFA, and PGC1α were obtained from Santa Cruz Biotechnology (Santa Cruz, CA, USA); TELO2, Cyclin D1, and γ-H2A.x were obtained from Abcam (Cambridge, UK); and p-mTOR, mTOR, p-ATM, ATM, p-p53 (ser15), MDM2, DEC1, p-c-JUN, c-JUN, p-Akt, Akt, p-p38, p38, p-PI3K, PI3K, p-ERK, ERK, p-Chk2, Chk2, p-AMPK, AMPK, p-eIF2α eIF2α, p-P70S6K, P70S6K, p-TSC2, TSC2, and 4EBP1 were obtained from Cell Signaling (Danvers, MA, USA).

### 4.6. Cell Cycle Profiles

The cell cycle profiles were assessed via propidium iodide (PI) staining to measure cellular DNA content. Cells were trypsinized and washed with PBS, and then resuspended in 1 mL PBS, fixed in 5 mL 70% ice-cold ethanol, and stored overnight at −30 °C. The following day, the cells were washed with ice-cold PBS containing 1% FBS, centrifuged, and stained with PI staining solution (5 μg/mL PI in PBS, 0.5% Triton X-100, and 0.5 μg/mL RNase A) for 30 min at 37 °C in the dark. The samples were analyzed using a FACSCalibur flow cytometer and Cell Quest Pro software, v 6.1 (BD Biosciences, Franklin Lakes, NJ, USA).

### 4.7. Telomerase Activity

To measure telomerase activity, cells were lysed in CHAPS lysis buffer, and protein concentration was determined using the DC protein assay. An equal amount of protein was added to the TRAP reaction mixture and incubated at 30 °C for 30 min. PCR amplification was carried out, and the products were resolved on a 10% non-denaturing polyacrylamide gel. The gel was stained with SYBR Gold, and the telomerase ladder was visualized using a UV transilluminator. Quantification was performed using ImageJ software, v 1.44a (NIH, Bethesda, MD, USA), and telomerase activity was expressed as the ratio of the intensity of the ladder to the internal control.

### 4.8. Invasion Assay

The invasion assay was conducted using Transwell chambers coated with Matrigel matrix (BD Biosciences). Cells were added to the upper chamber in serum-free DMEM, while DMEM containing 10% FBS was added to the lower chamber. After 16 h of incubation at 37 °C in a 5% CO_2_ incubator, non-migrated cells were removed from the upper chamber. The cells were fixed with 3.8% formaldehyde in PBS, stained with 0.1% crystal violet, and counted under a microscope (10× objective).

### 4.9. Wound-Healing Assay

To initiate the scratch wound healing assay, 3 × 10^5^ cells were seeded per well in a 24-well plate and allowed to incubate under 5% CO_2_ at 37 °C until a confluent monolayer formed (after 24 h). Using a sterile 200 μL pipette tip, a vertical cross was created in each well, and the cells were subsequently treated with different concentrations of tramadol. The closure of the scratch was monitored and captured at 0 h (immediately after wounding) and 16 h post-wounding using a LeadView 2800AC-FL microscope (equipped with a 40× objective) (Leader Scientific, New Taipei, Taiwan, ROC), and ImageJ (NIH) was used to determine the change in the wound area.

### 4.10. Reactive Oxygen Species (ROS) Assay

Intracellular ROS levels were measured using DCFH-DA staining. Cells were washed twice with PBS and incubated with 10 μM DCFH-DA in the dark at 37 °C for 30 min. Cells were then washed with PBS and analyzed using the FACSCalibur flow cytometer and Cell Quest Pro software, v 6.1 (BD Biosciences).

### 4.11. Apoptosis Assay

The PE Annexin-V apoptosis detection kit (BD Biosciences) with 7-AAD was employed to evaluate apoptosis, following the manufacturer’s instructions. The FACSCalibur flow cytometer and Cell Quest Pro software, v 6.1 (BD Biosciences) were utilized to measure the population of apoptotic stage.

### 4.12. Reverse Transcription–Polymerase Chain Reaction (RT–PCR)

The isolation of total RNA was performed using TRIzol reagent (Invitrogen, Waltham, MA, USA). MMLV reverse transcriptase (Epicentre Biotechnologies, Madison, WI, USA) was employed to carry out first-strand cDNA synthesis, utilizing 1 μg of total RNA at 37 °C for 60 min. The PCR reactions were run on a Veriti Thermal Cycler (Applied Biosystems, Carlsbad, CA, USA), and the PCR primer sequence and ID number are provided below (Table 1). These primers were synthesized and verified by Mission Biotech Co. Ltd. (Taipei, Taiwan, ROC). 

### 4.13. Statistical Analysis

The data were presented as mean ± SD, and the experiments were repeated independently three times. All group comparisons were conducted using Student’s *t*-tests, and statistical significance was established at *p* < 0.05.

## 5. Conclusions

Our data showed that TELO2 is involved in several functions of GBM cells, including cell cycle progression, EMT, ROS, apoptosis, and telomerase activity. The crosstalk between TELO2 and the responsiveness of TMZ or curcumin were mediated through the TELO2–TTI1–TTI2 complex, the p53-dependent complex, the mitochondrial-related complex, and signaling pathways in GBM8401 cells. Hence, our work provides new insight that TELO2 might modulate target proteins mediated through the complex of PIKKs in its involvement in cell cycle progression, EMT, and drug response in GBM patients.

## Figures and Tables

**Table 1:** PCR primers used in this study.

Gene ID	Gene Name	Primer Sequence (5′–3′)
9894	*TELO2*	Forward: 5′-TTTCGAGACTGCTGGGGAAC-3′ Reverse: 5′-TGAGGTCAGATAGTCGGCCA-3′
9675	*TTl1*	Forward: 5′-TGAGAGAAAGGCTGGAAGACG-3′ Reverse: 5′-ACCTTCCAGTGTGGGTGAAC-3′
80185	*TTl2*	Forward: 5′-GCTAGGTCCGGATCCTGTTAG-3′ Reverse: 5′-ATGGTTGCTCCAAGCTGTGT-3′
7157	*p53*	Forward: 5′-CTCTGACTGTACCACCATCCACTA-3′ Reverse: 5′-GAGTTCCAAGGCCTCATTCAGCTC-3′
1026	*p21*	Forward: 5′-CTGAGCCGCGACTGTGATGCG-3′ Reverse: 5′-GGTCTGCCGCCGTTTTCGACC-3′
8553	*DEC-1*	Forward: 5′-GTACCCTGCCCACATGTACC-3′ Reverse: 5′-GCTTGGCCAGATACTGAAGC-3′
467	*ATF3*	Forward: 5′-GAGGATTTTGCTAACCTGAC-3′ Reverse: 5′-TAGCTCTGCAATGTTCCTTC-3′
2194	*FAS*	Forward: 5′-TGAGCCTCATGCGCCTGGAC-3′ Reverse: 5′-CGCACCTCCTTGGCAAACAC-3′
9804	*TOM20*	Forward: 5′-GTGTATGCGGGGCCCTTTTC-3′ Reverse: 5′-ACATCATCTTCAGCCAAGCTCT-3′
7019	*mtTFA*	Forward: 5′-GCGTTTCTCCGAAGCATGTG-3′ Reverse: 5′-TTGTGCGACGTAGAAGATCC-3′
10891	*PGC1α*	Forward: 5′-GTAAATCTGCGGGATGATGG-3′ Reverse: 5′-ATTGCTTCCGTCCACAAAA-3′
3725	*c-JUN*	Forward: 5′-TGGGCACATCACCACTACAC-3′ Reverse: 5′-AGTTGCTGAGGTTGGCGTA-3′
2597	*GAPDH*	Forward: 5′-CTTCATTGACCTCAACTAC-3′ Reverse: 5′-GCCATCCACAGTCTTCTG-3′

## Data Availability

Our mRNA microarray data were deposited into the NCBI Gene Expression Omnibus (accession number GSE231503).
